# Mechanistic Perspectives of Maslinic Acid in Targeting Inflammation

**DOI:** 10.1155/2015/279356

**Published:** 2015-09-27

**Authors:** Wei Hsum Yap, Yang Mooi Lim

**Affiliations:** ^1^School of Biosciences, Taylor's University, No. 1, Jalan Taylor's, 47500 Subang Jaya, Selangor, Malaysia; ^2^Department of Preclinical Sciences, Faculty of Medicine and Health Sciences, Universiti Tunku Abdul Rahman, Lot PT 21744, Jalan Sungai Long, Bandar Sungai Long, 43000 Kajang, Selangor, Malaysia

## Abstract

Chronic inflammation drives the development of various pathological diseases such as rheumatoid arthritis, atherosclerosis, multiple sclerosis, and cancer. The arachidonic acid pathway represents one of the major mechanisms for inflammation. Prostaglandins (PGs) are lipid products generated from arachidonic acid by the action of cyclooxygenase (COX) enzymes and their activity is blocked by nonsteroidal anti-inflammatory drugs (NSAIDS). The use of natural compounds in regulation of COX activity/prostaglandins production is receiving increasing attention. In Mediterranean diet, olive oil and table olives contain significant dietary sources of maslinic acid. Maslinic acid is arising as a safe and novel natural pentacyclic triterpene which has protective effects against chronic inflammatory diseases in various *in vivo* and *in vitro* experimental models. Understanding the anti-inflammatory mechanism of maslinic acid is crucial for its development as a potential dietary nutraceutical. This review focuses on the mechanistic action of maslinic acid in regulating the inflammation pathways through modulation of the arachidonic acid metabolism including the nuclear factor-kappa B (NF-*κ*B)/COX-2 expression, upstream protein kinase signaling, and phospholipase A_2_ enzyme activity. Further investigations may provide insight into the mechanism of maslinic acid in regulating the molecular targets and their associated pathways in response to specific inflammatory stimuli.

## 1. Introduction

Maslinic acid is a natural pentacyclic triterpene which can be found in various natural sources including medicinal herbs [1, 2], edible vegetables and fruits [3, 4], especially in the skin of olives [5]. The amount of maslinic acid in table olives constitutes 0.8% in weight when extracted from the solid residues while the concentration in olive oil ranges from 38 mg/kg in extra virgin olive oil to 721 mg/kg in crude pomace olive oil [6]. Olives and olive oil are regular dietary components in the Mediterranean region which confers protection against chronic diseases. Daily consumption of approximately 40 g or 10 medium size olives corresponds to the intake of 28 mg maslinic acid per day [7]. It is hypothesized that this habitual consumption of olives and virgin olive oil will expose the intestinal epithelium to high concentrations of maslinic acid which confers health-protecting properties [8, 9]. On the other hand, maslinic acid can also be found in plants used for traditional Asian medicine, for example,* Eriobotrya japonica *[10, 11]*, Campsis grandiflora* [12],* Geum japonicum* [1], and* Agastache rugosa* [13], which are used to treat diverse inflammatory diseases. The pharmacological effects of maslinic acid have been reported in various experimental models including their antitumour [14, 15], anti-inflammatory [16, 17], cardioprotective [18], antiviral [19], antimalarial [20], neuroprotective [21], and antioxidant [22] activities. Considering its wide distribution in the plant kingdom and biological activities, it is suggested that maslinic acid is a natural and safe molecule. Maslinic acid has been assessed for its toxicity effects in animal models fed with high doses of this triterpene and it did not produce any signs of morbidity and mortality [23]. Recent investigation further showed that maslinic acid has weak inhibitory activities on cytochrome P450 (CYP) isoforms, suggesting that it has low potential to cause possible toxicity and drug interactions involving CYP enzymes [24].

### 1.1. Bioactive Properties of Pentacyclic Triterpenoids

Triterpenoids represent a group of compounds characterized by the 30-carbon isoprenoid skeleton molecule with over 100 distinct skeletons [25]. Triterpenoids are formed from cyclization of squalene or oxidosqualene. They may be acyclic, monocyclic, bicyclic, tricyclic, tetracyclic, pentacyclic, or hexacyclic [26]. Pentacyclic triterpenes are often bioactive (antitumor, antiviral, antidiabetic, and anti-inflammatory) and have huge therapeutic potential. There are numerous examples of enzymes that can be inhibited by pentacyclic terpenoids, indicating the ability of these compounds to act broadly in a nonspecific mode on multiple targets [27]. More importantly, pentacyclic triterpenoids scaffolds also have unique safety profiles [28]. For example, corosolic acid (antidiabetic) is already on the market and several other pentacyclic triterpenes are under clinical trials or ready to be launched in the market. The anti-inflammatory effects of pentacyclic triterpenoids are largely ascribed to their ability to inhibit molecular targets such as 5-lipoxygenase (LOX), inducible nitric oxide synthase (iNOS), cyclooxygenase (COX) 2, and nuclear factor-kappa B (NF-*κ*B) activities.

### 1.2. Structure-Activity Relationship between Pentacyclic Triterpenoids and Their Effects on Inflammation

**Figure 1:**
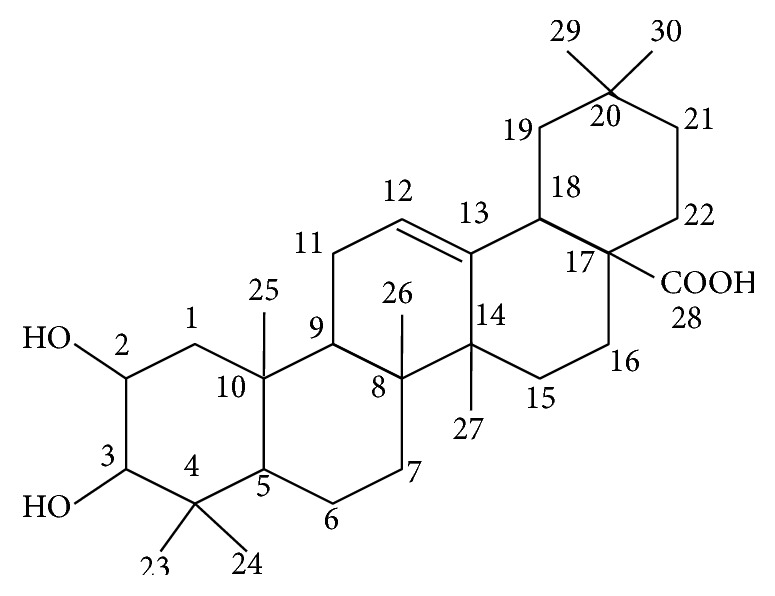
Molecular structure of maslinic acid.

**Figure 2:**
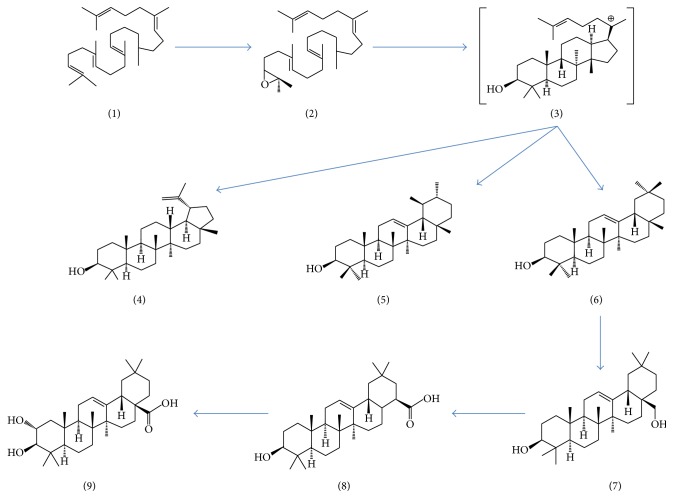
Biosynthesis of maslinic acid. Epoxidation of squalene (1) produces 2,3-oxidosqualene (2) which undergoes further cyclization into the dammarenyl cation (3). Dammarenyl cation undergoes D-ring expansion and additional cyclization to form products, such as lupeol (4), *α*-amyrin (5), and *β*-amyrin (6). Further oxidation steps convert *β*-amyrin to erythrodiol (7), followed by oleanolic acid (8) and finally maslinic acid (9).

Maslinic acid (Figure 1) is a pentacyclic triterpene compound. Pentacyclic compounds comprise the most numerous classes of oxidosqualene cyclases products [26]. Their structural variety reveals that they arise through a variety of cyclization modes. Maslinic acid is synthesized from the folding and cyclization of squalene (1) to oxidosqualene (2) and subsequently to the dammarenyl ring system. Dammarenyl (3) undergoes ring expansion and additional cyclization to form lupeol (4), *α*-amyrin (5), and *β*-amyrin (6) skeletons. Further oxidation steps convert *β*-amyrin to erythrodiol (7), followed by oleanolic acid (8) and finally maslinic acid (9) (Figure 2).

Several studies have shown that triterpenoids significantly suppress chronic inflammation. Pentacyclic triterpenoids with well-characterized biological activities include lupane, ursane, and oleanane type of compounds, such as lupeol, ursolic acid, and oleanolic acid. Structure-activity relationship study on the anti-inflammatory activities of triterpenoids showed that the basic carbon skeleton has no influence on the activity. The presence of C-28 or C-30 carboxylic group and an alcoholic group at C-28 increases the activity in carrageenan- and ethyl phenylpropiolate- (EPP-) induced edemas, respectively [29]. There are studies reporting that the presence of functional group at C-28 in triterpenic acids is capable of acting as hydrogen bond donors [30, 31]. On the other hand, it was shown that the oleanane skeleton (*β*-amyrin) is more potent than the ursane skeleton (*α*-amyrin) in inhibiting nitric oxide production induced by interferon-*γ* (IFN-*γ*) in mouse macrophages [32]. There are studies reporting that the presence of additional hydroxyl group at the C-2 position of maslinic acid confers antioxidant properties compared to oleanolic acid [16, 22]. However, it is suggested that the mechanism by which maslinic acid mediated inhibition of inflammatory cytokines may be independent of their antioxidant activity [33]. This review will highlight on the mechanism of maslinic acid in regulating inflammation, focusing on the arachidonic acid pathway.

## 2. The Arachidonic Acid Inflammatory Pathway

### 2.1. Inflammation: Homeostasis and Pathogenesis

Inflammatory response is initiated upon microbial infection and/or physical damage to restore the homeostatic balance of tissue structure and physiological function. Acute phase inflammation is a protective mechanism which involves coordinated actions of numerous molecular and cellular players. This process is characterized by migration of neutrophils and monocytes to the site of infection or injury, causing signs of inflammation such as swelling, pain, redness, and heat. Successful resolution of a typical acute phase inflammation requires elimination of the foreign microorganisms together with anti-inflammatory mediators that inhibit continuous recruitment of leukocytes. Persistent inflammatory response can lead to excessive tissue damage and loss of organ function. Hence, incomplete resolution of acute inflammation may predispose to chronic inflammation and autoimmunity disorders, such as multiple sclerosis, myocardial infarction, atherosclerosis, rheumatoid arthritis, stroke, Parkinson's disease, Alzheimer's disease, or cancer [34].

Local production of endogenous mediators at the site of infection and/or injury promotes the development of inflammation [35]. Prostaglandins and leukotrienes are potent mediators of inflammation derived from arachidonic acid (AA), a 20-carbon unsaturated fatty acid produced from membrane phospholipids. AA is released from the plasma membrane by phospholipase enzymes (mostly PLA_2_) which are activated by interleukin-8 (IL-8), microbial peptides, phagocytic particles, and nonspecific stimuli such as damage or injury [36]. Once AA is released, they can be metabolized into various C20 unsaturated lipids derivatives, collectively known as eicosanoids. Eicosanoids are formed via three main pathways, including the prostaglandins (PGs) and thromboxanes (TXs) (collectively termed prostanoids) formed by COX, leukotrienes (LTs) and lipoxins (LXs) by LOX, and epoxyeicosatrienoic acids (EETs) by cytochrome P450 enzymes [37–39].

### 2.2. Prostanoid Metabolism

**Figure 3:**
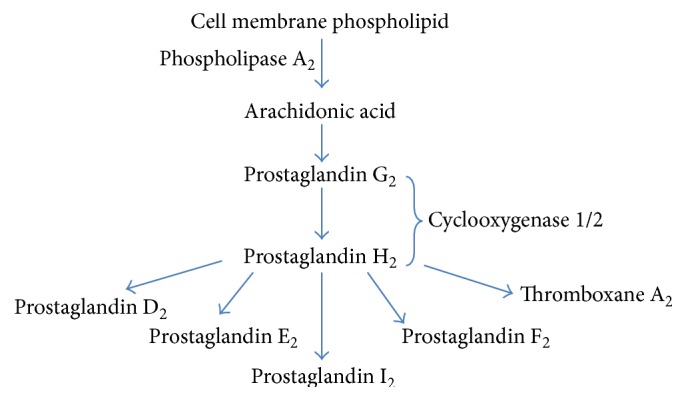
Prostanoid metabolism.

Prostanoids are synthesized by the cyclic pathway, which is initiated through the action of COX enzyme [40], also known as the prostaglandin G/H synthase (Figure 3). COX possesses both cyclooxygenase and peroxidase activities which catalyzes the two-step conversion of AA to PGG_2_ and then to PGH_2_ [41, 42]. It begins by catalyzing the bisoxygenation and cyclization of AA into hydroperoxy arachidonate metabolite PGG_2_ and is followed by the peroxidase element of the enzyme which reduces the hydroperoxide to its corresponding alcohol to form PGH_2_. There are two COX isoforms in humans, namely, COX-1 and COX-2. COX-1 is constitutively expressed in gastric mucosa, kidney, platelets, and vascular endothelial cells while COX-2 expression is inducible primarily at the site of inflammation, especially in macrophages and monocytes [43]. PGH_2_ is transformed into diverse forms of prostanoids through the action of prostaglandin D synthase, prostaglandin E synthase, prostaglandin F synthase, prostaglandin I synthase, and thromboxane A synthase, producing PGD_2_ [44], PGE_2_ [45], PGF_2_a [46], PGI_2_ [47], and TXA_2_ [48], respectively.

PGs play important roles in inflammatory response. PGE_2_ is one of the PGs which is of particular importance in regulating inflammation. PGE_2_ acts locally by binding to one or more of its respective receptors known as EP1–EP4 [49]. Under physiological conditions, PGE_2_ controls immune responses, blood pressure, gastrointestinal integrity, and fertility. Dysregulated PGE_2_ synthesis or degradation has been associated with a wide range of pathological conditions [50]. PGE_2_ enhances vasodilation and production of cAMP and decreases T-cell proliferation and lymphocyte migration and secretion of IL-1*α* and IL-2 [51, 52]. Knockout mouse studies further established the involvement of EP in inflammatory exudation [53]. PGE_2_ also mediates hyperalgesia through EP1 receptor signaling that plays important role in peripheral sensory neuronal signaling at the site of inflammation [54]. Other studies have also implicated the EP3 receptor in the inflammatory pain response mediated by low doses of PGE_2_ [55]. There are studies reporting that the release of PGE_2_ is mediated via activation of NF-*κ*B [56], a transcription factor which regulates numerous inflammatory genes and is considered as a possible target for therapeutic intervention. In fact, various studies have implicated NF-*κ*B in the transcriptional regulation of COX-2 [57, 58].

### 2.3. COX and Inflammation

Prostanoids as important mediators of inflammation are further supported by the fact that their biosynthesis is the target of nonsteroidal anti-inflammatory drugs (NSAIDS). NSAIDS such as ibuprofen, indomethacin, and aspirin all act upon the cyclooxygenase activity of both COX-1 and COX-2 enzymes. Both COX-1 and COX-2 may contribute to inflammatory response depending on the type of inflammatory stimulus and target tissue. It was shown that each isoform displays differential contribution to the development of inflammatory response depending on the experimental models used. For example, deletion of COX-2 inhibits synovial inflammation and joint destruction in collagen-induced arthritis model while COX-1-deficient mice showed no significant response [59, 60]. COX-1-derived PGs however contribute to development of arthritis in K/BxN serum-transfer arthritic model [61]. Specific COX-2 inhibitor was being developed because inhibition of COX-1 activity in the gut is associated with NSAID-induced ulcerations [62]. Targeted inhibition of COX-2 however increased the risk of cardiovascular disease [63]. It is reported that cardiovascular risk is dependent on the balance between vasodilating PGI2 and prothrombotic TXA2. These findings highlight the importance of pharmacological drug development in regulation of prostanoids profile.

## 3. Role of Maslinic Acid in Targeting Inflammation and Related Diseases

### 3.1. Inflammatory Modulating Effects of Maslinic Acid

Maslinic acid shows promising anti-inflammatory effects in several* in vivo* and* in vitro* experimental models. The anti-inflammatory effect of maslinic acid was first evaluated by Banno et al. in a model of 12-*O*-tetradecanoylphorbol-13-acetate- (TPA-) induced inflammation ear edema in mice [11]. Maslinic acid was applied topically to the tip of the mice ear 30 minutes before TPA treatment and the ear thickness was measured before treatment and 6 h after TPA treatment. Maslinic acid exhibited strong inhibitory effects (ID_50_ = 0.13 mg/ear) on TPA-induced inflammation. The same study also showed that triterpenes possessing more than one oxygen-bearing functional group such as both hydroxyl and carboxyl group have higher inhibitory activities on TPA-induced inflammation in mice. Maslinic acid which has one additional hydroxyl group compared to its parent compound oleanolic acid has lower ID50 for inhibition of inflammation.

Studies investigating the mechanism of maslinic acid in the* in vitro* models of inflammation showed that it regulates reactive species production and its corresponding inflammatory enzyme expressions. In a study evaluating the effect of maslinic acid in reactive oxygen and nitrogen-derived species and proinflammatory cytokines, it showed that maslinic acid significantly suppressed lipopolysaccharide- (LPS-) induced production of nitric oxide (NO) and iNOS gene expression, secretion of inflammatory cytokines interleukin-6 and tumour necrosis alpha (TNF-*α*), and the generation of hydrogen peroxide in murine peritoneal macrophages [16]. Qian et al. also observed that maslinic acid protects cortical neuron against oxygen-glucose deprivation-induced injury by inhibiting the level of NO, which was correlated with reduced iNOS protein and mRNA levels [21]. NO has a variety of regulatory mechanisms ranging from vasodilatation and blood pressure control to neurotransmission. High levels of NO produced from iNOS are an important mediator which lead to the production of reactive nitrogen oxide species, contributing to a wide range of chronic inflammations and infectious diseases [64]. Inhibition of NO production by blocking iNOS expression may be a strategy for treatment of chronic inflammation.

In another similar study, maslinic acid was shown to inhibit the expression of iNOS and COX-2 as well as the release of proinflammatory mediators including NO and TNF-*α* in LPS-induced cortical astrocyte cultures [17]. The inhibitory effects of COX-2 expression and enzyme activity by maslinic acid were also observed in several other culture systems including human macrophages, B lymphocytes, primary human chondrocytes, primary rat astrocytes, and SK-S-NH neuroblastoma type cell line [64]. The release of PGE_2_, an enzymatic product derived from COX-2, was also downregulated in primary human chondrocyte, primary rat astrocytes, and neuroblastoma type cell line. A thorough clinical study of patients with COX-2-related pathologies such as arthrosis, arthritis, or fibromyalgia reported the effectiveness of maslinic acid given in simple topical treatments in the affected areas, showing reduction of discomfort and considerable increase of flexibility of the joint [65]. Interestingly, complete remission of symptoms was observed in patients younger than 60 years old in less than one month of maslinic acid treatment. These findings collectively showed that maslinic acid has beneficial effects in modulating COX-2-related chronic inflammatory diseases.

### 3.2. Modulatory Effects of Maslinic Acid on Other Inflammation-Related Diseases

Inflammatory cells and cytokines also contribute to tumor growth and progression. Previously published literature has indicated that COX-2 creates a tumor-promoting environment which transforms epithelial cells. COX-2 is also inducible by oncogenes ras and scr, IL-1, hypoxia, ultraviolet light, epidermal growth factor, transforming growth factor-beta (TGF-*β*), and TNF-*α* [66]. Maslinic acid has been shown to inhibit the metastatic capacity of DU145 human prostate cancer cells. It reduces epidermal growth factor-induced DU145 cell migration via downregulation of both matrix metalloproteinases (MMPs) and urokinase-type plasminogen activator (uPA) systems. The study showed that maslinic acid inhibits hypoxia inducible factor-1*α* (HIF-1*α*), one of the regulators of angiogenesis in response to oxygen deficiency which has been associated with inducing expression of MMP-2, MMP-9, and uPA [67]. The protective effect of maslinic acid was also reported in a spontaneous intestinal polyposis animal model. The results showed that maslinic acid-enriched diet inhibited the formation of polyps in the small intestines of Apc^Min/+^ mice by regulating genes associated with inflammation pathways. It is suggested that maslinic acid suppress chronic inflammation which contributed to the development and sustainability of intestinal adenomatous polyps in Apc^Min/+^ mice [15].

## 4. Molecular Targets of Maslinic Acid in Regulating Inflammatory Pathway

Considering that maslinic acid regulates inflammation through inhibiting iNOS and COX-2 expression, it is postulated that it inhibits the activity of NF-*κ*B, a transcription factor which binds to the promoter sequence of these two enzymes. Li et al. proved that maslinic acid affects the NF-*κ*B pathway by inhibiting I*κ*B*α* phosphorylation, thus preventing NF-*κ*B translocation to nucleus and its DNA-binding activity to the COX-2 promoter in pancreatic cancer cells, Panc-28 [14]. NF-*κ*B is recognized as a stress-regulated transcription factor, which plays a key role in the control of inflammatory responses [68]. NF-*κ*B transcription factors are dimer proteins (typically p65/p50 heterodimer) retained in the cytosol by inhibitory proteins, including inhibitory-kappa B (I*κ*B) proteins I*κ*B*α*, I*κ*B*β*, I*κ*B*ε*, and I*κ*B*γ* [69]. After receiving a stimulatory signal such as LPS or TNF-*α*, the I*κ*B*α* inhibitory protein is phosphorylated by I*κ*B kinases (IKKs), which allow NF-*κ*B to translocate from the cytoplasm to the nucleus, where it binds to the promoter region and transcribes its target genes [70]. The inhibitory effect of maslinic acid on NF-*κ*B activation was also shown in Raji B lymphoma cells where it was correlated to the inhibition of COX-2 expression in a concentration-dependent manner. In addition, the authors also showed that maslinic acid was able to suppress activation of activator protein (AP-1) [71].

The NF-*κ*B transcriptional activity can be modulated through phosphorylation by various members of the mitogen-activated protein kinase (MAPK) family. Majority of the studies investigated the effect of maslinic acid in mediating apoptosis via activation of JNK and p38 MAPK [72, 73]. Meanwhile, others have shown that maslinic acid inhibited osteoclastogenesis by downregulating phosphorylation of MAPKs and AP-1 activity, inhibited the I*κ*B*α* phosphorylation and degradation, and blocked NF-*κ*B phosphorylation, nuclear translocation, and DNA-binding activity by downregulating receptor activator of NF-*κ*B (RANK) expression and blocking RANK interaction with tumor necrosis factor receptor-associated factor 6 (TRAF6) [74]. Maslinic acid also suppressed the expression of PKC *β*I, *δ*, and *ζ* in tumour promoter phorbol 12-myristate 13-acetate- (PMA-) induced Raji cell model [75]. The authors proposed that the inhibition of PKC activity by maslinic acid may explain the regulation of downstream targets in the signaling cascade including NF-*κ*B and downstream gene expression such as COX-2, VEGF, cyclin D1, and MMP-9 [14, 75]. Other natural triterpenoids such as *α*-amyrin have been reported to inhibit PMA-induced mouse skin inflammation through suppressing PKC*α* [76].  Table 1 summarizes the various inflammatory modulating effects of maslinic acid.

A recent study showed that phospholipase A_2_ (PLA_2_) may be a potential binding target of maslinic acid [20]. PLA_2_ is responsible for hydrolyzing membrane phospholipids and releasing AA which serves as a substrate COX-2-mediated prostaglandins production [77]. There is evidence that AA release also occurs through different PKC isoforms activation [78]. Inhibition of PLA_2_ would decrease formation of AA and production of PGs. PLA_2_ inhibitors are currently being developed as a therapeutic strategy, focusing on the development of specific PLA_2_ isoform inhibitors for treatment of inflammatory diseases. Mammalian tissues contain many secretory PLA_2_s (sPLA_2_s) including group I/II/V/X [79]. The enzyme sPLA_2_-GV has been involved in prostanoids production in inflammatory cells such as macrophages and mast cells [80, 81]. A preliminary study by Yap et al. showed that maslinic acid inhibited sPLA_2_-GV enzyme activity in a concentration-dependent manner. The sPLA_2_-GV enzyme inhibitory activity shown by maslinic acid is more potent compared to ursolic acid. Molecular docking study further showed that maslinic acid binds to the sPLA_2_-GV interfacial phospholipid binding site via hydrogen bonding and hydrophobic interaction, thereby inhibiting the enzymatic activity of sPLA_2_-GV [82].

## 5. Future Prospects

**Figure 4:**
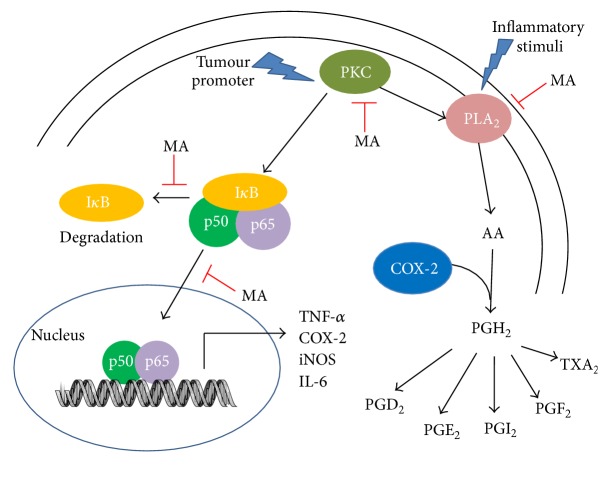
Molecular mechanism of maslinic acid in targeting inflammatory pathways. PKC plays a central role in the activation of NF-*κ*B (p50/p65). Once activated, the I*κ*B protein is degraded which allows NF-*κ*B to translocate from cytoplasm to the nucleus, where it transcribes the expression of downstream proinflammatory genes such as TNF-*α*, COX-2, iNOS, and IL-6. It was shown that maslinic acid inhibited PKC activation, I*κ*B*α* degradation, and NF-*κ*B nuclear translocation, which might correlate to its anti-inflammatory properties. In addition, evidence also demonstrated the role of PKC in mediating PLA_2_ phosphorylation and AA release. Once released from membrane phospholipids, AA can be converted into prostanoids through the action of COX enzymes. Considering that prostanoids are important mediators of inflammation, the anti-inflammatory effect of maslinic acid may be explained through its effect in inhibiting PKC activation and/or PLA_2_ enzyme activity which reduces the substrate availability for COX-2-mediated prostanoids biosynthesis in inflammatory cells.

Maslinic acid has been widely accepted as a natural compound with anti-inflammatory effects. Recent studies have elucidated its molecular mechanism and potential binding targets (Figure 4). Nevertheless, it is still unknown how maslinic acid regulates the PKC/NF-*κ*B inflammatory signaling pathways which contribute to the inhibition of iNOS/COX-2 activity and NO/PGE_2_ release. Further studies are required to unravel the mechanism of maslinic acid in regulating upstream protein kinases or transcriptional targets in response to specific inflammatory stimuli which leads to the reduction of proinflammatory enzyme expression and activity. Direct interaction and inhibition of the PLA_2_ enzyme upstream the arachidonic acid pathway may also be one of the mechanisms of maslinic acid which reduces substrate availability for COX-2-mediated PGE_2_ formation. It is hoped that future studies will provide insight into the anti-inflammatory mechanism of maslinic acid and further develop maslinic acid as a potential dietary nutraceutical.

## Figures and Tables

**Table 1:** Inflammatory modulating effect of maslinic acid.

Inflammatory model	Modulatory effect of maslinic acid	References
12-*O*-Tetradecanoylphorbol-13-acetate- (TPA-) induced ear edema	Maslinic acid reduced TPA-induced ear edema at the concentration of 0.13 mg per ear	[11]

Spontaneous intestinal polyposis animal model	Maslinic acid-enriched diet inhibited the formation of polyps in the small intestines of Apc^Min/+^ mice by regulating genes associated with inflammation pathways	[15]

Lipopolysaccharide- (LPS-) induced murine macrophages	Maslinic acid suppressed production of nitric oxide (NO) and inducible nitric oxide synthase (iNOS) gene expression, secretion of inflammatory cytokines interleukin-6, and tumour necrosis alpha (TNF-*α*)	[16]

LPS-induced cortical astrocyte cultures	Maslinic acid inhibits the expression of iNOS and COX-2 as well as the release of proinflammatory mediators including NO and TNF-*α*	[17]

Oxygen-glucose deprivation-induced cortical neuron injury	Maslinic acid reduced NO levels and iNOS mRNA and protein expression	[21]

COX-2-related pathologies such as arthrosis, arthritis, or fibromyalgia	Maslinic acid given in simple topical treatments showed reduction of discomfort and considerable increase of flexibility of the joint	[65]

Phorbol 12-myristate 13-acetate- (PMA-) induced Raji B lymphoma cells	Maslinic acid suppresses PKC *β*I, *δ*, and *ζ*, COX-2 expression, NF-*κ*B, and AP-1 activation	[71, 75]

Osteoclastogenesis and bone loss	Maslinic acid suppresses osteoclastogenesis by regulating receptor activator of NF-*κ*B ligand- (RANKL-) mediated NF-*κ*B and mitogen-activated protein kinase (MAPK) signaling pathways	[74]

## References

[1] Xu H.-X., Zeng F.-Q., Wan M., Sim K.-Y. (1996). Anti-HIV triterpene acids from *Geum japonicum*. *Journal of Natural Products*.

[2] Kim D.-H., Han K.-M., Chung I.-S. (2005). Triterpenoids from the flower of *Campsis grandiflora* K. Schum. as human acyl-CoA: cholesterol acyltransferase inhibitors. *Archives of Pharmacal Research*.

[3] Kalogeropoulos N., Chiou A., Ioannou M., Karathanos V. T., Hassapidou M., Andrikopoulos N. K. (2010). Nutritional evaluation and bioactive microconstituents (phytosterols, tocopherols, polyphenols, triterpenic acids) in cooked dry legumes usually consumed in the Mediterranean countries. *Food Chemistry*.

[4] Yin M.-C., Lin M.-C., Mong M.-C., Lin C.-Y. (2012). Bioavailability, distribution, and antioxidative effects of selected triterpenes in mice. *Journal of Agricultural and Food Chemistry*.

[5] Romero C., García A., Medina E., Ruíz-Méndez M. V., Castro A. D., Brenes M. (2010). Triterpenic acids in table olives. *Food Chemistry*.

[6] Pérez-Camino M. C., Cert A. (1999). Quantitative determination of hydroxy pentacyclic triterpene acids in vegetable oils. *Journal of Agricultural and Food Chemistry*.

[7] Bianchi G., Pozzi N., Vlahov G. (1994). Pentacyclic triterpene acids in olives. *Phytochemistry*.

[8] Yang M., Guang J. W., Su J. W. (2005). Quantitative analysis of 23-hydroxybetulinic acid in mouse plasma using electrospray liquid chromatography/mass spectrometry. *Rapid Communications in Mass Spectrometry*.

[9] Juan M. E., Wenzel U., Ruiz-Gutierrez V., Daniel H., Planas J. M. (2006). Olive fruit extracts inhibit proliferation and induce apoptosis in HT-29 human colon cancer cells. *The Journal of Nutrition*.

[10] Lu H., Xi C., Chen J., Li W. (2009). Determination of triterpenoid acids in leaves of *Eriobotrya japonica* collected at in different seasons. *Zhongguo Zhong Yao Za Zhi*.

[11] Banno N., Akihisa T., Tokuda H. (2005). Anti-inflammatory and antitumor-promoting effects of the triterpene acids from the leaves of *Eriobotrya japonica*. *Biological and Pharmaceutical Bulletin*.

[12] Kim D.-H., Han K.-M., Chung I.-S. (2005). Triterpenoids from the flower of *Campsis grandiflora* K. Schum. as human Acyl-CoA: cholesterol acyltransferase inhibitors. *Archives of Pharmacal Research*.

[13] Zou Z. M., Cong P. Z. (1991). Studies on the chemical constituents from roots of *Agastache rugosa*. *Yao Xue Xue Bao*.

[14] Li C., Yang Z., Zhai C. (2010). Maslinic acid potentiates the anti-tumor activity of tumor necrosis factor *α* by inhibiting NF-*κ*B signaling pathway. *Molecular Cancer*.

[15] Sánchez-Tena S., Reyes-Zurita F. J., Díaz-Moralli S. (2013). Maslinic acid-enriched diet decreases intestinal tumorigenesis in ApcMin/+ mice through transcriptomic and metabolomic reprogramming. *PLoS ONE*.

[16] Márquez Martín A., de la Puerta Vázquez R., Fernández-Arche A., Ruiz-Gutiérrez V. (2006). Supressive effect of maslinic acid from pomace olive oil on oxidative stress and cytokine production in stimulated murine macrophages. *Free Radical Research*.

[17] Huang L., Guan T., Qian Y. (2011). Anti-inflammatory effects of maslinic acid, a natural triterpene, in cultured cortical astrocytes via suppression of nuclear factor-kappa B. *European Journal of Pharmacology*.

[18] Hussain Shaik A., Rasool S. N., Kareem M. A., Krushna G. S., Akhtar P. M., Devi K. L. (2012). Maslinic acid protects against isoproterenol-induced cardiotoxicity in albino Wistar rats. *Journal of Medicinal Food*.

[19] Parra A., Rivas F., Lopez P. E. (2009). Solution- and solid-phase synthesis and anti-HIV activity of maslinic acid derivatives containing amino acids and peptides. *Bioorganic & Medicinal Chemistry*.

[20] Moneriz C., Mestres J., Bautista J. M., Diez A., Puyet A. (2011). Multi-targeted activity of maslinic acid as an antimalarial natural compound. *FEBS Journal*.

[21] Qian Y., Guan T., Tang X. (2011). Maslinic acid, a natural triterpenoid compound from *Olea europaea*, protects cortical neurons against oxygen-glucose deprivation-induced injury. *European Journal of Pharmacology*.

[22] Montilla M. P., Agil A., Navarro M. C. (2003). Antioxidant activity of maslinic acid, a triterpene derivative obtained from *Olea europaea*. *Planta Medica*.

[23] Sánchez-González M., Lozano-Mena G., Juan M. E., García-Granados A., Planas J. M. (2013). Assessment of the safety of maslinic acid, a bioactive compound from *Olea europaea* L.. *Molecular Nutrition and Food Research*.

[24] Sun M., Tang Y., Ding T., Liu M., Wang X. (2015). Investigation of cytochrome P450 inhibitory properties of maslinic acid, a bioactive compound from *Olea europaea* L., and its structure-activity relationship. *Phytomedicine*.

[25] Connolly J. D., Hill R. A. (2002). Triterpenoids. *Natural Product Reports*.

[26] Xu R., Fazio G. C., Matsuda S. P. T. (2004). On the origins of triterpenoid skeletal diversity. *Phytochemistry*.

[27] Glinski J., Branly K. L. Pentacyclic triterpenes.

[28] Sporn M. B., Liby K. T., Yore M. M., Fu L., Lopchuk J. M., Gribble G. W. (2011). New synthetic triterpenoids: potent agents for prevention and treatment of tissue injury caused by inflammatory and oxidative stress. *Journal of Natural Products*.

[29] Del Carmen Recio M., Giner R. M., Máñez S., Ríos J. L. (1995). Structural requirements for the anti-inflammatory activity of natural triterpenoids. *Planta Medica*.

[30] Ziegler H. L., Franzyk H., Sairafianpour M. (2004). Erythrocyte membrane modifying agents and the inhibition of *Plasmodium falciparum* growth: structure-activity relationships for betulinic acid analogues. *Bioorganic and Medicinal Chemistry*.

[31] Prades J., Vögler O., Alemany R. (2011). Plant pentacyclic triterpenic acids as modulators of lipid membrane physical properties. *Biochimica et Biophysica Acta—Biomembranes*.

[32] Syrovets T., Büchele B., Gedig E., Slupsky J. R., Simmet T. (2000). Acetyl-boswellic acids are novel catalytic inhibitors of human topoisomerases I and II*α*. *Molecular Pharmacology*.

[33] Marquez-Martin A., De La Puerta R., Fernandez-Arche A., Ruiz-Gutierrez V., Yaqoob P. (2006). Modulation of cytokine secretion by pentacyclic triterpenes from olive pomace oil in human mononuclear cells. *Cytokine*.

[34] Ricciotti E., Fitzgerald G. A. (2011). Prostaglandins and inflammation. *Arteriosclerosis, Thrombosis, and Vascular Biology*.

[35] Stables M. J., Gilroy D. W. (2011). Old and new generation lipid mediators in acute inflammation and resolution. *Progress in Lipid Research*.

[36] Piper P., Vane J. (1971). The release of prostaglandins from lung and other tissues. *Annals of the New York Academy of Sciences*.

[37] Serhan C. N., Hamberg M., Samuelsson B. (1984). Trihydroxytetraenes: a novel series of compounds formed from arachidonic acid in human leukocytes. *Biochemical and Biophysical Research Communications*.

[38] Samuelsson B., Dahlen S.-E., Lindgren J. A., Rouzer C. A., Serhan C. N. (1987). Leukotrienes and lipoxins: structures, biosynthesis, and biological effects. *Science*.

[39] Capdevilla J. H., Falck J. R., Dishman E., Karara A. (1990). Cytochrome P-450 arachidonate oxygenase. *Methods in Enzymology*.

[40] Smyth E. M., Grosser T., Wang M., Yu Y., FitzGerald G. A. (2009). Prostanoids in health and disease. *Journal of Lipid Research*.

[41] Hamberg M., Samuelsson B. (1973). Detection and isolation of an endoperoxide intermediate in prostaglandin biosynthesis. *Proceedings of the National Academy of Sciences of the United States of America*.

[42] Nugteren D. H., Hazelhof E. (1973). Isolation and properties of intermediates in prostaglandin biosynthesis. *Biochimica et Biophysica Acta*.

[43] Dubois R. N., Abramson S. B., Crofford L. (1998). Cyclooxygenase in biology and disease. *The FASEB Journal*.

[44] Morrow J. D., Parsons W. G., Roberts L. J. (1989). Release of markedly increased quantities of prostaglandin D2 in vivo in humans following the administration of nicotinic acid. *Prostaglandins*.

[45] Tanaka Y., Ward S. L., Smith W. L. (1987). Immunochemical and kinetic evidence for two different prostaglandin H-prostaglandin E isomerases in sheep vesicular gland microsomes. *Journal of Biological Chemistry*.

[46] Hayashi H., Fujii Y., Watanabe K., Urade Y., Hayaishi O. (1989). Enzymatic conversion of prostaglandin H2 to prostaglandin F(2*α*) by aldehyde reductase from human liver: comparison to the prostaglandin F synthetase from bovine lung. *Journal of Biological Chemistry*.

[47] Moncada S., Gryglewski R., Bunting S., Vane J. R. (1976). An enzyme isolated from arteries transforms prostaglandin endoperoxides to an unstable substance that inhibits platelet aggregation. *Nature*.

[48] Ullrich V., Haurand M. (1983). Thromboxane synthase as a cytochrome P450 enzyme. *Advances in Prostaglandin, Thromboxane, and Leukotriene Research*.

[49] Trebino C. E., Stock J. L., Gibbons C. P. (2003). Impaired inflammatory and pain responses in mice lacking an inducible prostaglandin E synthase. *Proceedings of the National Academy of Sciences of the United States of America*.

[50] Legler D. F., Bruckner M., Uetz-von Allmen E., Krause P. (2010). Prostaglandin E_2_ at new glance: novel insights in functional diversity offer therapeutic chances. *International Journal of Biochemistry & Cell Biology*.

[51] Lewis A. J., Nelson D. J., Sugrue M. F. (1975). On the ability of prostaglandin E_1_ and arachidonic acid to modulate experimentally induced oedema in the rat paw. *British Journal of Pharmacology*.

[52] Kaley G., Hintze T. H., Panzenbeck M., Messina E. J. (1985). Role of prostaglandins in microcirculatory function. *Advances in Prostaglandin, Thromboxane, and Leukotriene Research*.

[53] Hata A. N., Breyer R. M. (2004). Pharmacology and signaling of prostaglandin receptors: multiple roles in inflammation and immune modulation. *Pharmacology & Therapeutics*.

[54] Moriyama T., Higashi T., Togashi K. (2005). Sensitization of TRPVI by EP_1_ and IP reveals peripheral nociceptive mechanism of prostaglandins. *Molecular Pain*.

[55] Minami T., Nakano H., Kobayashi T. (2001). Characterization of EP receptor subtypes responsible for prostaglandin E_2_-induced pain responses by use of EP_1_ and EP_3_ receptor knockout mice. *British Journal of Pharmacology*.

[56] Catley M. C., Chivers J. E., Cambridge L. M. (2003). IL-1 beta-dependent activation of NF-kappa B mediates PGE_2_ release via the expression of cyclooxygenase-2 and microsomal prostaglandin E synthase. *FEBS Letters*.

[57] Yamamoto K., Arakawa T., Ueda N., Yamamoto S. (1995). Transcriptional roles of nuclear factor *κ*B and nuclear factor-interleukin-6 in the tumor necrosis factor *α*-dependent induction of cyclooxygenase-2 in MC3T3-E1 cells. *Journal of Biological Chemistry*.

[58] D'Acquisto F., Iuvone T., Rombolà L., Sautebin L., Di Rosa M., Carnuccio R. (1997). Involvement of NF-kappaB in the regulation of cyclooxygenase-2 protein expression in LPS-stimulated J774 macrophages. *FEBS Letters*.

[59] Ochi T., Ohkubo Y., Mutoh S. (2003). Role of cyclooxygenase-2, but not cyclooxygenase-1, on type II collagen-induced arthritis in DBA/1J mice. *Biochemical Pharmacology*.

[60] Myers L. K., Kang A. H., Postlethwaite A. E. (2000). The genetic ablation of cyclooxygenase 2 prevents the development of autoimmune arthritis. *Arthritis & Rheumatism*.

[61] Chen M., Boilard E., Nigrovic P. A. (2008). Predominance of cyclooxygenase 1 over cyclooxygenase 2 in the generation of proinflammatory prostaglandins in autoantibody-driven K/BxN serum-transfer arthritis. *Arthritis & Rheumatism*.

[62] Mardini I. A., FitzGerald G. A. (2001). Selective inhibitors of cyclooxygenase-2: a growing class of anti-inflammatory drugs. *Molecular Interventions*.

[63] Funk C. D., FitzGerald G. A. (2007). COX-2 inhibitors and cardiovascular risk. *Journal of Cardiovascular Pharmacology*.

[64] Zamora R., Vodovotz Y., Billiar T. R. (2000). Inducible nitric oxide synthase and inflammatory diseases. *Molecular Medicine*.

[65] Osuna P. Use of maslinic acid for the treatment of diseases and the symptoms thereof by means of cox-2 inhibition.

[66] Fosslien E. (2000). Molecular pathology of cyclooxygenase-2 in neoplasia. *Annals of Clinical and Laboratory Science*.

[67] Park S. Y., Nho C. W., Kwon D. Y., Kang Y.-H., Lee K. W., Park J. H. Y. (2013). Maslinic acid inhibits the metastatic capacity of DU145 human prostate cancer cells: possible mediation via hypoxia-inducible factor-1*α* signalling. *British Journal of Nutrition*.

[68] Gilmore T. D. (2006). Introduction to NF-kappaB: players, pathways, perspectives. *Oncogene*.

[69] Chen F. E., Ghosh G. (1999). Regulation of DNA binding by Rel/NF-*κ*B transcription factors: structural views. *Oncogene*.

[70] Yamamoto Y., Gaynor R. B. (2001). Therapeutic potential of inhibition of the NF-*κ*B pathway in the treatment of inflammation and cancer. *The Journal of Clinical Investigation*.

[71] Hsum Y. W., Yew W. T., Hong P. L. V. (2011). Cancer chemopreventive activity of maslinic acid: suppression of COX-2 expression and inhibition of NF-*κ*B and AP-1 activation in raji cells. *Planta Medica*.

[72] Reyes-Zurita F. J., Pachón-Peña G., Lizárraga D., Rufino-Palomares E. E., Cascante M., Lupiáñez J. A. (2011). The natural triterpene maslinic acid induces apoptosis in HT29 colon cancer cells by a JNK-p53-dependent mechanism. *BMC Cancer*.

[73] Wu D.-M., Zhao D., Li D.-Z., Xu D.-Y., Chu W.-F., Wang X.-F. (2011). Maslinic acid induces apoptosis in salivary gland adenoid cystic carcinoma cells by Ca^2+^-evoked p38 signaling pathway. *Naunyn-Schmiedeberg's Archives of Pharmacology*.

[74] Li C., Yang Z., Li Z. (2011). Maslinic acid suppresses osteoclastogenesis and prevents ovariectomy-induced bone loss by regulating RANKL-mediated NF-*κ*B and MAPK signaling pathways. *Journal of Bone and Mineral Research*.

[75] Mooi L. Y., Yew W. T., Hsum Y. W., Soo K. K., Hoon L. S., Chieng Y. C. (2012). Suppressive effect of maslinic acid on PMA-induced protein kinase C in human B-lymphoblastoid cells. *Asian Pacific Journal of Cancer Prevention*.

[76] Medeiros R., Otuki M. F., Avellar M. C. W., Calixto J. B. (2007). Mechanisms underlying the inhibitory actions of the pentacyclic triterpene *α*-amyrin in the mouse skin inflammation induced by phorbol ester 12-O-tetradecanoylphorbol-13-acetate. *European Journal of Pharmacology*.

[77] Murakami M., Nakatani Y., Atsumi G.-I., Inoue K., Kudo I. (1997). Regulatory functions of phospholipase A_2_. *Critical Reviews in Immunology*.

[78] Xu J., Weng Y.-I., Simoni A. (2002). Role of PKC and MAPK in cytosolic PLA_2_ phosphorylation and arachadonic acid release in primary murine astrocytes. *Journal of Neurochemistry*.

[79] Schaloske R. H., Dennis E. A. (2006). The phospholipase A_2_ superfamily and its group numbering system. *Biochimica et Biophysica Acta: Molecular and Cell Biology of Lipids*.

[80] Han S.-K., Yoon E. T., Cho W. (1998). Bacterial expression and characterization of human secretory class V phospholipase A_2_. *Biochemical Journal*.

[81] Reddy S. T., Winstead M. V., Tischfield J. A., Herschman H. R. (1997). Analysis of the secretory phospholipase A_2_ that mediates prostaglandin production in mast cells. *The Journal of Biological Chemistry*.

[82] Yap W. H., Laweeza Meetoo B. I., Ahemad N. Elucidating the molecular mechanisms of interaction between maslinic acid and human group V-secreted phospholipase A2 (hGV-sPLA2).

